# Factors associated with older people's independent living from the viewpoint of health and functional capacity: a register‐based study

**DOI:** 10.1002/nop2.39

**Published:** 2015-11-03

**Authors:** Anette Ahlqvist, Hanna Nyfors, Riitta Suhonen

**Affiliations:** ^1^ Department of Nursing ScienceUniversity of Turku20014TurkuFinland; ^2^ Ministry of Social Affairs and Health00031 Government00023HelsinkiFinland

**Keywords:** Functional capacity, health, independent living, older people, register‐based study

## Abstract

**Aim:**

The aim of this study was to identify factors associated with independent living of older people from the point of view of health and functional capacity.

**Background:**

Living independently at home is major wish for older people but is depending on health and functional capacity among others. Objective and subjective assessments have been considered important in determining threats for independent living but both of these views have rarely included in the same study.

**Design:**

Descriptive, cross‐sectional register‐based study was conducted.

**Methods:**

Data were collected using the Health and Functional Capacity survey by identifying the factors of health examinations of a cohort (*N *=* *292) of 75‐year old's, born in 1936 (*N *=* *388), in one Finnish medium‐sized municipality in 2011. This study is part of the Functional Ageing project Kaste 2013. The data were analysed statistically by using descriptive analysis, cross‐tabulation and logistic regression.

**Results:**

Partly, different factors were associated with subjective and objective health and functional capacity showing wide range of individuality. Worsening subjective health was associated with worsened self‐assessment of life situation. Worsening subjective health threatens independent living. Factors statistically significantly associated with worsening subjective health were low physical activity, falls during the last 6 months, not managing heavy housework, being sometimes lonely or dejected, having diagnosed diseases or health problems and polypharmacy.

## Introduction

Living independently at home is considered the main wish of the majority of older people in European countries (Harrefors *et al*. 2009, Stula 2012). At the moment, most older people do live at home and that is what most European societies aim to support, for example, by implementing support programmes (e.g. Stula 2012, European Union 2014, The Kings Fund 2014). It seems that the majority of older people in Europe, especially in early older age, are in good condition and capable of independent living (Wahrendorf & Siegrist 2010). However, some older people need support to live independently (Eloranta *et al*. 2008) and this need should be recognized rapidly and organized comprehensively around the individual's needs and not around single symptoms or diseases (The Kings Fund 2014). Therefore, healthcare professionals in every contact with older people need to evaluate older people's individual situation in the broad sense to support independent living from their point of view. This includes health, functional capacity, resources, personal attributes, living circumstances and environment. Health and functional capacity are the most influential parts of independent living (e.g. Bravell *et al*. 2007, Beswick *et al*. 2010). Information provided by the cohort register‐based studies is useful in planning the support, care and services for ageing population to support healthy ageing, independence and self‐care.

## Background

**Figure 1:**
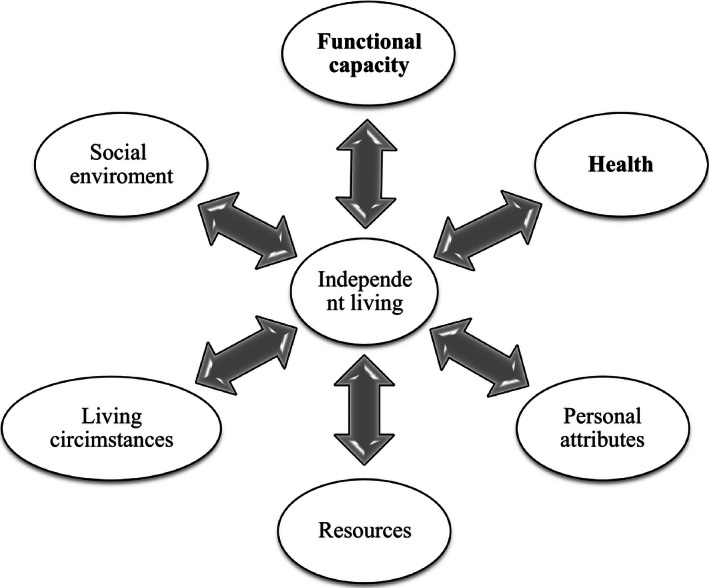
Factors associated with independent living based on previous studies (the main interests in this study are highlighted).

Older people's independent living has been studied in different fields of science. Health sciences and medicine have focused on objectively assessing activities of daily living (ADL) (Secker *et al*. 2003). In nursing science, independent living has been studied for example through social function (Arve *et al*. 2009) and personal resources (Eloranta *et al*. 2008). Previous studies (e.g. Secker *et al*. 2003, Bravell *et al*. 2007, Beswick *et al*. 2010) have identified factors associated with independent living, supporting or threatening it (Figure 1).

It is scientifically proven that living independently at home requires a certain state of health and functional capacity (Bravell *et al*. 2007, Beswick *et al*. 2010). Functional capacity was defined in terms of the ability to perform ADL (Secker *et al*. 2003, Bravell *et al*. 2007). Health and functional capacity can be assessed objectively as well as subjectively. Health was defined in terms of complete physical, mental and social well‐being (WHO 2015). Objective assessment health typically includes for example chronic diseases, medication use, malnutrition, measurement of blood pressure, vision, cognitive status, smoking and alcohol use (see Table 1). Subjective health is considered more as an emotional state (Bravell *et al*. 2007). Although objective health and ability to perform ADL can weaken as one become older, subjective health can remain at good level for much longer (Bravell *et al*. 2007). Subjectively assessed good health and functional capacity have been found to support independent living (Yuen *et al*. 2007, Beswick *et al*. 2010).

**Table 1:** Factors that are associated with and can threaten independent living based on previous studies

Factors that are associated with and can threaten independent living based on previous studies.	Previous studies
Weakening of functional capacity and health	Mastrian (2001), Bravell *et al*. (2007), Yuen *et al*.(2007), Beswick *et al*. (2010)
Disability in ADL	Mastrian (2001), Bravell *et al*. (2007), Yuen *et al*.(2007), Beswick *et al*. (2010)
Chronic diseases	Plath (2008), Beswick *et al*. (2010)
Loneliness	Eloranta *et al*. (2008), Plath (2008), Arve *et al*. (2009)
Polypharmacy	Plath (2008), Beswick *et al*. (2010)
Isolation	Plath (2008)
Immobility	Beswick *et al*. (2010)
Insecurity	Plath (2008)
Falls	Mahoney and Goc (2009), Beswick *et al*. (2010)
Malnutrition	Kloseck *et al*. (2010)
Being a caregiver	Arve *et al*. (2009)
Unsuitable type of accommodation	Mahoney and Goc (2009)

Independent living is also associated with one's personal resources (Secker *et al*. 2003, Dunèr & Nordström 2005, Eloranta *et al*. 2008, Plath 2008), living circumstances (Mahoney & Goc 2009), social environment (Secker *et al*. 2003, Plath 2008, Arve *et al*. 2009, Beswick *et al*. 2010) and personal attributes (Yuen *et al*. 2007, Eloranta *et al*. 2008). Personal resources are an important part of being capable of independent living (Secker *et al*. 2003, Dunèr & Nordström 2005, Eloranta *et al*. 2008, Plath 2008). Also relatives’ resources count (Dunèr & Nordström 2005). Social environment and meaningful relationships are found to be associated with independent living and can support it (Secker *et al*. 2003, Yuen *et al*. 2007, Plath 2008, Arve *et al*. 2009, Larsson *et al*. 2009, Beswick *et al*. 2010). Social relationships can weaken the capability of living at home (Plath 2008, Beswick *et al*. 2010), for example in the case of caregivers taking care of an invalid spouse (Arve *et al*. 2009). Older people feel that a socially useful role (Yuen *et al*. 2007, Plath 2008), acceptance and respect (Plath 2008) support their independent living. In concrete terms, independent living can be supported by economic factors (Yuen *et al*. 2007, Bennett *et al*. 2010) and with various technological solutions (Larsson *et al*. 2009, Piau *et al*. 2014). However, there is limited research on the use of technology in frail older people living at home (Piau *et al*. 2014).

On the basis of previous studies some factors can be determined that are associated with and can threaten independent living of older people (Table 1) when occurring in older persons’ lives. These factors can lead to a situation where the home is no longer a safe and suitable place for the older person. Weakening of functional capacity and health, or experience of it, can lead to difficulties in ADL (Mastrian 2001, Bravell *et al*. 2007, Yuen *et al*.^2007^, Beswick *et al*. 2010). Disability in ADL is considered as the main reason why independent living can come under threat. For example, chronic diseases, especially heart and vascular diseases, diabetes and dementia and polypharmacy associated with them can weaken the ability to perform ADL and therefore threaten independent living (Plath 2008, Beswick *et al*. 2010). Loneliness (Eloranta *et al*. 2008, Plath 2008, Arve *et al*. 2009), isolation (Plath 2008), immobility (Beswick *et al*. 2010), insecurity (Plath 2008), falls (Mahoney & Goc 2009, Beswick *et al*. 2010), malnutrition (Kloseck *et al*. 2010), being an informal caregiver (Arve *et al*. 2009) and unsuitable type of accommodation (Mahoney & Goc 2009) can also be factors that are associated with and can threaten independent living.

There can be individual (Dunèr & Nordström 2005) and cultural (Bennett *et al*. 2010) differences in factors associated with independent living of older people. There are probably more factors that are associated with or can threaten independent living than those mentioned here. Despite some studies that have focused on describing and naming factors associated with independent living of the older people, the phenomenon has not yet been fully studied. Studies are often conducted with a small sample and the results cannot be generalized. However, healthcare professionals often assess older people's capability of living at home and try to support independent living through associated factors. That is why associated factors should be more precisely identified. Identification of associated factors is also important because it enables proper allocation of health services.

In our study, living independently at home means living alone or with a spouse in one's own or rented apartment without any regular professional assistance. Living independently at home requires a certain state of health and functional capacity, which are the main interests in our study.

## Aim

The aim of this study was to identify factors associated with independent living of older people from the point of view of subjective and objective health and functional capacity based on health examination. Functional capacity and health in old age have been found to be associated with independent living (Bravell *et al*. 2007, Beswick *et al*. 2010). The research questions were:

- What do health examinations reveal of 75‐year olds’ independent living from the point view of subjective and objective health and functional capacity?
- What factors are associated with 75‐year olds’ subjective and objective health and functional capacity and therefore independent living?

## Methods

### Study design, settings and sample

This descriptive, cross‐sectional register‐based study is part of the Kaste Programme (2013), Functional Ageing project. Data in our study were register data from one city. The study site was one medium‐sized Finnish municipality with about 40,000 inhabitants. The whole cohort of independently living 75‐year‐old people, born in 1936 and living independently at home in the area of one municipality, comprised 388 persons (total sample). Inclusion criteria were; (1) a 75‐year old in year 2011; and (2) living independently at home. Excluded were persons who: (1) lived in nursing homes or hospitals; or (2) had regular professional assistance. Those fulfilling the inclusion criteria, as assessed by the public health nurse, received an invitation to participate in the health examination. Data recorded in the register included information and measurements of 292 persons (75% of the total amount of those fulfilling the inclusion criteria) who were willing to participate in the health examination conducted by the public health nurse in 2011.

### Procedures

Permission for using the register data in this study was requested and given by the register holder and the manager of health care and social services of the municipality where this study took place. Based on the Finnish regulations register based anonymous data can be used for research purposes (Finnish Personal Data Act 22.4.1999/523). The data were collected in primary healthcare centre by one public health nurse and her registrations were noted in the patients’ records. All those fulfilling the inclusion criteria received the questionnaire by mail from the public health nurse together with the invitation to participate in the health examination and conversation. Completed questionnaires and the public health nurse's registration of the results of the health examinations were the register data used in this study.

### Measures

Data were collected using the Health and functional capacity – survey including structured and open‐ended questions about socio‐demographic variables, functional capacity, health and living situation before attending the health examinations (Table 2). The questionnaire was developed by a group of local clinical experts in the Functional Ageing project based on the national application form of the Association of Finnish Local and Regional Authorities. The chair of the developmental group was a local director of older people care and services and the experts in the group included a head physician, head nurse, occupational therapist, registered nurse, primary health nurse, specialist in elderly care and a project manager.

**Table 2:** The list of variables and descriptive statistics

What the associated factor measures	All variables used	Independent variable	*n*	Frequency categories (%)
	*Socio‐demographic data*			
	1. Gender	X	292	Male (43); female (57)
	2. Marital status		292	Single (3); married or in a relationship (73); divorced (6); widow (18)
	3. Type of accommodation		292	Town house (51); row house (15); multi‐storey building (34)
	4. Living arrangements		291	Living alone (26); living with spouse (72); living with someone else (2)
	*Functional capacity*			
Disability in ADL	5. Type of accommodation suitable for functional capacity	X	271	Yes (91); no (9)
	6. Way of travelling from a place to another		292	
	6a. Walking			Yes (53); no (47)
	6b. Bicycle			Yes (47); no (53)
	6c. Own car			Yes (76); no (24)
	6d. Someone else's car			Yes (13); no (87)
	6e. Public transport			Yes (9); no (91)
	6f. Other way			Yes (3); no (97)
	7. Driving a car	X	289	Yes (64); no (36)
Immobility	8. Prevalence of physical activity	X	286	Every day (67); rarely or never (33)
	9. Using stairs		292	Yes (80); no (20)
	10. Cause of not using stairs		55	Pain in the knee (42); other pain (13); Impaired mobility (5); other (40)
Falls	11. Falls during last 6 months	X	287	Yes (18); no (82)
	12. Cause of falling		53	Slippery (23); dizziness (9); Loss of balance (9); other (58)
Disability in ADL	13. Managing of light housework		291	Yes (92); no (8)
	14. Managing of heavy housework	X	290	Yes (67); no (33)
	15. Managing of home repairs		291	Yes (76); no (24)
	16. Managing of shopping and banking		291	Yes (93); no (7)
Isolation	17. Participation in activities outside the home	X	286	Yes (64); no (36)
	18. Hobbies	X	286	Yes (91); no (9)
Loneliness	19. Loneliness/dejection	X	292	No (63); sometimes (35); often (2)
	20. Using the Internet		251	Yes (35); no (65)
Objective functional ability	21. Public health nurse's assessment of functional capacity		279	Good/moderate (78); impaired (22)
	*Health*			
Subjective health (self‐report)	22. Self‐assessed life situation		289	Good/satisfying (90); poor (10)
	23. Subjective health		291	Good (40); satisfying (55); poor (5)
Weakening of health and functional capacity	24. Subjective health compared to last year	X	288	Better/similar (82); worse (18)
Chronic diseases	25. Diagnosed diseases/health problems	X	292	Yes (92); no (8)
	26a. Heart or vascular diseases	X	269	Yes (65); no (35)
	26b. Musculoskeletal disorder	X	269	Yes (47); no (53)
	26c. Diabetes	X	269	Yes (20); no (80)
	26d. Lung disease	X	269	Yes (9); no (91)
	26e. Cancer	X	269	Yes (11); no (89)
	26f. Mental disorder	X	269	Yes (7); no (93)
	26g. Other disease/health problem	X	269	Yes (76); no (24)
	27. Regular medicine use	X	292	Yes (90); no (10)
Polypharmacy	28. Number of medicine used	X	286	0–4 (70); 5 or more (30)
	29. Sleeping situation		280	Well (63); poorly (21); with the help of medication (16)
Malnutrition	30. Appetite		289	Good (96); poor (4)
	31. Does health status disturb sexuality?		188	No (52); slightly (25); a lot (23)
Physical measurement	32. BMI	X	283	Under 30 (72); 30 or over (28)
	33. Systolic blood pressure	X	283	Under 140 (27); 140 or over (73)
	34. Diastolic blood pressure	X	283	Under 90 (84); 90 or over (16)
	35. Eyesight		238	Normal (48); impaired (5); guided to further testing (17);regularly tested (30)
	36. Hearing		289	Normal (72); impaired (20); hearing aid in use (8)
	37. Cognitive Memory status	X	228	Impaired (6); no actions done (94)
	38. Memory test done		292	Yes (17); no (83)
	39. Eating a proper meal daily		291	Yes (96); no (4)
	40. Smoking	X	292	Yes (8); no (92)
	41. Alcohol use	X	288	Never or once a month (63); more than once a month (37)
Objective health assessment	42. Public health nurse's assessment of the results of health examination		286	Healthy 75‐year old (57); risk of decline in functional capacity (43)

First, the participants were asked a self‐report about their functional capacity and health. Concerning functional capacity (14 questions)*,* the participants were asked about their living and social circumstances (five questions), physical activity (2), way of travelling from one place to another (2), how they are able to perform their daily activities (4) and about falls and cause of possible falls (1) (Table 2). Response scale for the structured questions was dichotomous yes or no and for the open‐ended a word or sentence. The public health nurse used a completed questionnaire as basis for the discussion in the health encounter. Based on the discussion, the public health nurse assessed if the type of accommodation was suitable for the functional capacity and made assessment of functional capacity.

Concerning health (three questions), participants were asked how they would rate their health and life situation and compare it to the previous year. For example, they were asked how do they feel about their health situation and the answering scale was very good, good, moderate and poor. In addition, there were questions using a dichotomous scale yes and no concerning sleeping, nutrition, sexuality, smoking and alcohol use, one of each. With an open‐ended question participants were asked if they had any chronic diseases (seven different chronic diseases, yes/no), or regular medicine use. In discussion they were also asked about sensory function (eyesight and hearing) and cognitive/memory status. Cognitive status was assessed during the discussion with the public health nurse and MMSE was used for those having some difficulties in communicating their health in the encounter.

Second, the public health nurse made physical measurements of blood pressure and body mass index (BMI). Based on the results of health examination the public health nurse assessed if the older people were healthy or at risk of decline in functional capacity forming the objective view of health and functional capacity (two assessments).

We studied relationships with factors which, based on previous studies, were associated with independent living. The dependent variables were public nurse's assessment of functional capacity (variable 21), self‐assessed life situation (22), subjective health (23) and public nurse's assessment of the results of health examination (42). We selected these variables as dependent because they best describe subjective (variables 22, 23) and objective (21, 42) functional capacity and health in this data set (see Table 2). Independent variables were selected based on researchers knowledge of factors associated with older people's health and functional capacity.

### Data analysis

The data were analysed statistically by using the SPSS version 20 IBM SPSS Statistics Standard version 20 (IBM Inc, New York, USA). Descriptive statistics, cross‐tabulation and logistic regression were used. First, a description of the 75‐year‐old people who participated in health examinations was computed by using descriptive statistics, such as frequencies, means and standard deviations. Second, relationships between the dependent and independent variables were examined using cross‐tabulation and logistic regression. Those independent variables which correlated statistically significantly with dependent variables in cross‐tabulation were entered into the logistic regression. A dichotomous logistic regression was used with variables having only two categories, while cumulative logistic regression was used with variables with three or more categories. We wanted to explain dependent variables with several independent variables. To succeed we need to capsulize some variable categories because of the frequency rate. To interpret logistic regression we used odds ratios with 95% confidence intervals. If the confidence intervals of the odds ratio included 1 there were no statistically significant relationships among the variables. A value of *P *<* *0·05 was regarded as statistically significant. As statistical significance test, we used chi‐square or Fisher's exact test. (Polit & Beck 2004.)

### Ethical considerations

Based on the Finnish Personal Data Act (22.4.1999/523) informed consent of ‘participants’ is not required in register‐based studies and they do not need to be informed about the study.

However, it is still ethically challenging that the older people did not know about this study when they came to the health examination. At the time it was not known that their registration would end up as study data. In this case, we trusted the register holder's (the manager of health care and social services) assessment about using registration in this study enacted in the Finnish legislation (Act on the Openness of Government Activities 1999/621 §28). Ethical approval was not sought as the register holder assesses also ethical issues while granting permission to use the data for research purposes based on the written study plan. As this was a register‐based data collected from health examinations in health care and used secondary registered data, informed consent from individuals (*n* = 292) was not possible afterwards. Although the older people did not know that their registration was used as study data, their privacy was extremely important and anonymity was maintained throughout the study. The public health nurse removed all identification information from the data and it was handed over to the research group without revealing the identity of the older people (Finnish Personal Data Act 22.4.1999/523). During data analysis, the researcher kept the data out of reach of outsiders. After analysis study, data were returned to the organization which had given permission to use it in this study. Confidentiality was maintained by following RCR guidelines (‘Responsible conduct of research and procedures for handling allegations of misconduct in Finland’) (Finnish Advisory Board on Research Integrity 2013).

## Results

### Socio‐demographic characteristics

Altogether, 75% (*n* = 292) of the cohort participated in the health examination and their register data were used in this study. Most of them were women (57%), married or in a relationship (73%) and living with a spouse (72%) in a townhouse (Table 2).

### Subjective and objective health and functional capacity

Health examinations revealed that most participants were in good condition and capable of living independently. More than half of the participants were assessed by the public health nurse as being healthy and not at risk of decline in functional capacity. The public health nurse assessed the 75‐year‐olds’ functional capacity as mostly good (63%) or moderate (15%). The 75‐year‐olds’ self‐assessed life situation (90%) and subjective (95%) health were also mostly good or satisfying although nearly all (92%) had some chronic diseases or health problems. Most of the respondents were physically active (67%) and did not feel lonely or dejected (63%). The majority had hobbies (91%) or participated in activities outside the home (64%). For the majority, type of accommodation was suitable for their functional capacity (91%). The 75‐year olds mostly managed light (92%) and heavy (67%) housework, home repairs (76%), banking and shopping (93%) independently.

### Factors associated with 75‐year‐olds’ independent living

When assessing capability to live independently, it is important to understand what factors are associated with independent living. In this study, we viewed independent living from the perspective of subjective and objective health and functional capacity and studied factors associated to them.

Subjectively assessed health and functional capacity were associated with partly the same factors as objectively assessed health and functional capacity, such as chronic diseases, number of medicines used, BMI and systolic blood pressure. The quality of disease played a role. For example, objective health and functional capacity were associated with having cancer and mental disorders, but not with subjective assessment of health and functional capacity. Smoking was associated with objective view, but not when assessing health and functional capacity subjectively. Participating in activities outside the home and physical activity were associated with subjective and objective health and functional capacity and therefore with independent living. The ability to perform ADL had a similar association with both. According to the results, memory status was statistically significantly associated with subjective health.

To identify factors that are associated and can threaten 75‐year‐olds’ independent living, we studied, using logistic regression, why the participants self‐assessed their life situation or subjective health as poor, as well as why they assessed their functional capacity as impaired, or why they were assessed as being at risk of decline in functional capacity (Table 3).

**Table 3:** Logistic regression for factors associated statistically significantly with 75‐year‐olds’ subjective and objective health and functional capacity. Comparison for impaired conditions

Dependent variables representing independent living	Variable outcome	Associated factor (independent variable)	Variable outcome	*P* value	Odds ratio	95% confidence interval
Self‐assessed life situation	Poor	Subjective health compared to last year	Worse	<0·0001	15·543	4·474–54·002
Subjective health	Poor	Prevalence of physical activity	Rarely or never	0·0375	2·062	1·043–4·076
		Falls during last 6 months	Yes	0·0018	3·730	1·635–8·510
		Managing of heavy housework	No	<0·0001	4·913	2·275–10·610
		Loneliness/dejection	Sometimes	0·0101	2·326	1·222–4·427
		Diagnosed diseases/health problems	Yes	0·0069	5·980	1·634–21·881
		Number of medicines used	5 or more	0·0021	3·089	1·506–6·334
Public health nurse's assessment of functional capacity	Impaired	Is type of accomodation suitable for functional capacity?	No	0·0287	7·606	1·235–46·846
		Managing of heavy housework	No	<0·0001	13·388	4·534–39·535
		Subjective health compared to last year	Worse	0·0093	4·353	1·437–13·187
		Number of medicines used	5 or more	0·0006	6·069	2·164–17·021
		BMI	30 or over	0·0232	3·458	1·185–10·091
		Smoking	Yes	0·0128	7·612	1·541–37·599
Public health nurse's assessment of the results of health examination	Risk of decline in functional capacity	Managing of heavy housework	No	0·0003	4·336	1·943–9·675
		Loneliness/dejection	Sometimes	0·0042	2·973	1·410–6·267
		Subjective health compared last year	Worse	0·0023	4·784	1·748–13·088
		Number of medicines used	5 or more	0·0025	3·305	1·524–7·170
		BMI	30 or over	0·0004	4·354	1·921–9·869

Experiencing subjective health as worsening during the last year explained statistically significantly why the 75‐year olds assessed their life situation as poor. Low physical activity, falls during the last 6 months, not managing heavy housework, being sometimes lonely or dejected, having diagnosed diseases/health problems and polypharmacy explained statistically significantly why the 75‐year olds assessed their subjective health as poor.

Type of accommodation not being suitable, not managing heavy housework, experiencing subjective health worsening during last year, having polypharmacy, being obese or being smoker explained statistically significantly why public health nurse assessed functional capacity of the 75‐year olds as impaired. Not managing heavy housework, being sometimes lonely or dejected, experiencing subjective health worsening during last year, having polypharmacy or being obese explained statistically significantly why public health nurse assessed the 75‐year olds as being at risk of decline in functional capacity.

## Discussion

The main aim of this study was to identify factors associated with independent living from the point of view of health and functional capacity. The results revealed that most of the 75‐year olds were in good condition from a subjective and objective point of views and capable of continuing independent living. It is noticeable that partly different factors were associated with subjective and objective health and functional capacity. This means that healthcare professionals may have different views of the factors that are associated with and can threaten independent living of older people than the older people themselves. This study highlights that healthcare professionals should take into account older persons’ subjective views on their life situation, health and functional capacity. This is very important when assessing the capability of living at home or trying to support it. In previous studies older persons’ personal point of view has been found to be more important from the perspective of independent living than objective view (Bravell *et al*. 2007, Bennett *et al*. 2010).

In our study, older people experienced subjective health worsening if certain factors occurred in their lives. Worsening health led to worsening assessment of life situation. As found in earlier studies, worsening subjective health is associated with and can threaten independent living (Mastrian 2001, Yuen *et al*. 2007, Beswick *et al*. 2010). Factors that explain health worsening can therefore also be factors that may threaten independent living (Mastrian 2001, Beswick *et al*. 2010). Older people did feel their subjective health as worsening if they could not manage heavy housework, they had a fall during the last 6 months, their physical activity was low, they were sometimes lonely or dejected, had diagnosed diseases or health problems or polypharmacy. Earlier studies found out that falls (Mahoney & Goc 2009, Beswick *et al*. 2010), feelings of loneliness (Eloranta *et al*. 2008, Plath 2008, Arve *et al*. 2009) and having chronic diseases (Plath 2008, Beswick *et al*. 2010) were associated with inability to manage at home supporting our results.

Not managing heavy housework refers to difficulties in activities of daily living. Based on previous studies, this has been identified as a factor which is associated with and can threaten independent living (Mastrian 2001, Bravell *et al*. 2007, Yuen *et al*. 2007, Beswick *et al*. 2010). In this study, prevalence of physical activity was associated with both subjective and objective health and functional capacity. If the older person's physical activity was low, it was associated with worsening health. This result is new although immobility has been found to be a factor that can threaten independent living (Beswick *et al*. 2010).

Feelings of loneliness or dejection were also associated with why older persons felt their health to be worsening. A third of the 75‐year olds sometimes felt lonely or dejected. This is an important finding as it has been identified as a risk factor for hospital admission and hospitalization (Stevens *et al*. 2014) and therefore can threaten independent living (Eloranta *et al*. 2008, Plath 2008, Arve *et al*.^2009^, Larsson *et al*. 2009). Various kinds of chronic diseases and regular medications were common among the older people. Chronic diseases (Plath 2008, Larsson *et al*. 2009, Beswick *et al*. 2010, Kloseck *et al*. 2010) and polypharmacy (Mahoney & Goc 2009, Beswick *et al*. 2010) can threaten independent living.

Certain factors led to a situation where the older people's objective health and functional capacity were assessed as being impaired. These factors were unsuitable type of accommodation, not managing heavy housework, being sometimes lonely or dejected and experiencing subjective health worsening during the preceding year, having polypharmacy, being obese or being a smoker. Smoking and BMI were not associated with subjective health and functional capacity; otherwise similar factors were associated with both.

### Limitations

In interpreting the results, some limitations need to be taken into account. This study viewed independent living from the point of views of subjective and objective health and functional capacity. Doing so, the researchers not only selected a narrow scope but also concentrated on the most important (Yuen *et al*. 2007, Beswick *et al*. 2010) factors, health and functional capacity. Therefore, future research is needed to investigate factors associated with independent living, such as living circumstances and social environment. The strength of this study was the use of total amount of those 75‐year‐old people living at home in the area of one municipality. The measurement was based on the older persons’ subjective responses to a series of questions asked and registered by public health nurse during the health encounters, physical measurement of blood pressure and BMI and objective assessment of health and functional capacity. The weakness of the study is the infrequent use of the standard assessment of cognitive status but was assessed during the health encounter decreasing full potential of the objectively assessed health situation. The questionnaire used included mainly dichotomous (yes/no) or three level scales (good, satisfying, poor) for reporting their health condition or functional capacity. The variables were single items and do not form any sum‐variables or similar making it impossible to assess reliability of the measure statistically. The questionnaire included content and determinants of independent living of older people found from the literature supporting content validity (Figure 1). The results of this study are not highly generalizable, but they can be used when designing more extensive studies. The results can be used in designing comprehensive instruments for evaluating health, functional capacity and independent living of older people in a variety of homes.

Earlier studies have reported smaller samples. However, the sample in this study was from one city in Finland and no generalizations can be made. Furthermore, it has to be observed that the data were not originally collected for study purposes and included some problems in coherent registrations and observations, weakening the validity of the data. In addition, some information was missing. The registrations were completed by one healthcare professional, making the observations systematic and accurate. However, it might weaken the external validity of the study. In addition, some important known factors remained outside the scope of this study, such as feelings of insecurity, which has been found to threaten independent living of older people (e.g. Plath 2008). Based on the registration this topic was discussed but not registered systematically and this information could not be used in the study. Also nutrition remains little studied although malnutrition has been determined to be a threatening factor (Kloseck *et al*. 2010) especially in people with memory disorders.

## Conclusions

When allocating health services for older people or trying to support independent living it is important to pay attention to subjective assessments of health, functional capacity and factors associated to them. Older persons’ expertise of their own situation and self‐management has to be highlighted. Older people's preventive healthcare services need to be developed so that individual differences are observed. There is variation in how much people want and are able to assign responsibility of their health to healthcare professionals. Not only paying attention to the medical conditions improvement of health literacy of older people but also to manage their differing health situations is needed (Health Literacy Europe 2014). The results are useful in nursing education as it is important to point out older persons’ individuality and to learn new ways and methods to promote their health in the home context. More preventive perspective and information delivery is needed in healthcare services, because older persons in European countries are likely to be in even better condition in the future than at present. As independent living of older people is important, accurate assessment is needed for planning services provided at home for the rapidly ageing population.

### Relevance to clinical practice

Based on the results, certain objective and subjective assessments of health and functional capacity are worth of exploring in every health encounter and healthcare contact with older people. Focus on single disease or symptom is not appropriate but the whole situation and circumstances of the individual older person need to be taken into account in facilitating independent living. First line nurses are in a position, where this kind of assessment takes place and enables also proactive nursing interventions to be planned.

## Conflict of interest

The authors state there is no conflict of interest.

## Author contributions

All authors have agreed on the final version and meet at least one of the following criteria [recommended by the ICMJE (http://www.icmje.org/recommendations/)]:

- substantial contributions to conception and design, acquisition of data or analysis and interpretation of data;
- drafting the article or revising it critically for important intellectual content.
